# Chemical composition, in vitro antioxidant, anticholinesterase, and antidiabetic potential of essential oil of *Elaeagnus umbellata* Thunb

**DOI:** 10.1186/s12906-021-03228-y

**Published:** 2021-02-22

**Authors:** Nausheen Nazir, Muhammad Zahoor, Faheem Uddin, Mohammad Nisar

**Affiliations:** 1 Department of Biochemistry, University of Malakand, Chakdara Dir (L), Khyber Pakhtunkhwa Pakistan; 2 Department of Electrical Engineering, Sarhad University of Science and Information Technology, Peshawar, Pakistan; 3 Department of Botany, University of Malakand, Chakdara Dir (L), Khyber Pakhtunkhwa Pakistan

**Keywords:** GC-MS, DPPH, ABTS, Anti-cholinesterase, α-Glucosidase and α-amylase

## Abstract

**Background:**

*Elaeagnus umbellata* Thunb. (autumn olive) is a high valued medicinal plant. It belongs to *Elaeagnaceae* family and is widely distributed in Himalayan regions of Pakistan. In the present study essential oil were extracted from the fruit of this plant and their antioxidant, anticholinesterase and antidiabetic potentials were also evaluated.

**Methods:**

Essential oils were extracted from the fruit of *E. umbellata* using hydro-distillation method and were characterized by GC-MS. The extracted oil were tested for its antioxidant, anticholinesterase, and antidiabetic potentials using standard protocols.

**Results:**

About 68 compounds were identified by GC-MS. The extracted oil exhibited a fairly high free radical scavenging activities against DPPH and ABTS radicals with IC_50_ values of 70 and 105 μg/mL respectively (for ascorbic acid, used as standard, the IC_50_ values were 32 and 29 μg/mL, respectively against the mentioned radicals). The essential oil also exhibited anticholinesterase activities with IC_50_ values of 48 and 90 μg/mL respectively against AChE and BChE (for galantamine used as standard, the IC_50_ values were 25 and 30 μg/mL respectively). The essential oil also exhibited antidiabetic potential with IC_50_ values of 120 and 110 μg/mL respectively against α-glucosidase and α-amylase (IC_50_ values for standard acarbose = 28 and 30 μg/mL respectively).

**Conclusion:**

Essential oil extracted from the fruits of *E. umbellata* exhibited reasonable antioxidant, anticholinesterase, and antidiabetic potentials that could be used as alternative medicine in treating diabetes and neurodegenerative disorders. However, further studies are needed to isolate responsible compounds and evaluate the observed potential in animal models.

**Graphical abstract:**

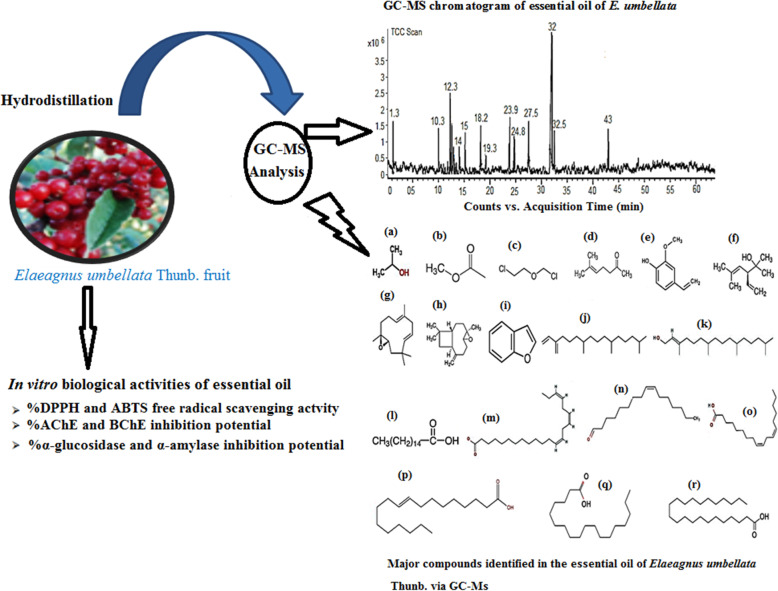

**Supplementary Information:**

The online version contains supplementary material available at 10.1186/s12906-021-03228-y.

## Background

Medicinal plants are considered as the essential part of human civilization since about 80% of the world population relies on medicinal plants in a number of health complications even in this modern era. Pakistan has a variety of land (plane, mountainous and desert) where a variety of plants grow, having medicinal importance [1]. Medicinal plants are valued much as they are factories of natural products and are widely used to treat various diseases. They produces a variety phytochemicals like; carotenoids, phenolic acids, phenols and flavonoids that have exhibited effective antioxidant properties along with other biological potentials [2]. The history of medicinal herb usage dates back to the distant past, many centuries and civilizations ago. Plants had played an important role in the treatment of various diseases since the beginning of human life on earth. However, the utilization of medicinal plants are usually limited to a particular area where they grow. Also the commercial utilization of high valued medicinal plants are discouraging one which is due to the unavailability of adequate scientific information about their medicinal uses [3].

Free radicals of oxygen and nitrogen are continuously produced during metabolism in animal and human bodies. They are very reactive, however, human body can efficiently detoxify them within seconds. Sometime reactive oxygen species are produced in larg quantities that harms the biologically important molecules leading to pathological conditions like stroke, rheumatoid arthritis, diabetes, inflammation, aging, cancer, and neurological disorders [4]. Any substance that can scavenge the reactive oxygen species is known as antioxidant. Due to resonance stabilization effect in benzene rings, phenolic compounds can effectively scavenge the free radicals [5]. Literature studies have revealed that the use of polyphenolic compounds present in fruits, tea, and vegetables can effectively minimize the risk of the mentioned diseases [6]. The most prevalent neurodegenerative disorder is Alzheimer^’^s disease (AD) characterized by low level of cholinergic transmission, deposition of β-amyloid, and increased oxidative stress [7]. This cholinergic deficit is due to degradation of neurotransmitters acetylcholine (ACh) by two enzymes acetyl cholinesterase (AChE) and butyryl cholinesterase (BChE). Inhibition of these two enzymes are used as strategy to maintain the level of ACh in body which would consequently relieve the symptoms associated with AD, Dementia and Parkinson^’^s disease [8]. The consumption of edible plants/vegetables throughout the world has enormously increased as they are chief sources of phenolic compounds and even out of the five recommended drugs used for the treatment of AD, two are plant phytochemicals [9].

Berry fruits are rich sources of phenolic compounds. Among berry fruit plants, *Elaeagnus umbellata* Thunb. is a member of the *Elaeagnaceae* family with a high medicinal value that is native to Southern Europe and Central Asia [10]. It is abundantly found in Himalayan regions of Pakistan as well [11]. The *Elaeagnus species* are traditionally used as antioxidant, anticancer, antinociceptive, anti-inflammatory, anti-mutagenic, anti-ulcerogenic, antimicrobial, antidiabetic, and neuroprotective agents [12–14]. Previously we have evaluated the antioxidant, antidiabetic, and anticholinesterase potential of different extracts and isolated compounds (rutin, epigallocatechin gallate, epigallocatechin, quercetin, morin, ellagic acid, catechin, chlorogenic acid, and pyrogallol) of *E. umbellata* [13, 14]. The floral volatiles and biological activities of *E. umbellata* and *E. angustifolia L.* have been reported [15–18]. A review of the literature revealed that no previous studies have been performed on the essential oil of fruit of *E. umbellata.*

Keeping in view the high medicinal importance of *E. umbellata* fruit the phytochemical composition of essential oil was determined through GC-MS. The extracted oil were also evaluated for their antioxidant, anticholinesterase, and antidiabetic potentials.

## Methods

### Chemicals and reagents

All the chemical used were of analytical grade. DPPH, ABTS, ascorbic acid, galantamine (*Lycoris Sp.*), potassium phosphate buffer (pH 8.0), acetylcholinesterase (*Electron eel* type-VI-S), butyrylcholinesterase (from aquine), acetylcholine iodide, and butyrylcholine iodide were obtained from Sigma-Aldrich, Switzerland. DTNB (5,5-dithio-bis-2-nitrobenzoic acid), 3, 5- Di nitro-salicylic acid, Type I α-glucosidase (*Saccharomyces cerevisiae*), Type VI α-amylase (porcine pancreas), PNPG (*p*-nitrophenyl-α- D-glucopyranose), and acarbose were obtained from Sigma-Aldrich, Germany.

### Plant material collection

*E. umbellata* Thunb. fruits were collected from the hilly areas of Kalam, Malakand Division, Khyber Pakhtunkhwa, Pakistan, in September, 2016. The plant sample was identified by plant taxonomist; Prof. Mehboob-ur-Rahman, P.G.C. Swat, Khyber Pakhtunkhwa, Pakistan. The plant specimens were deposited in the Botanical Garden Herbarium, University of Malakand, Pakistan with voucher number BGH.UOM.154. The plant variety selected was a wild one therefore, permission was taken from Divisional Forest Officer, Kalam and Local administration.

### Essential oil extraction

Essential oil from the fruits of *E. umbellata* were extracted through hydro distillation method using a Clevenger type apparatus connected with a condenser [19]. Distillation process was continued for 3 days at 100 °C, and the fruit volatile oils, yellowish in color were collected in glass bottles. Anhydrous sodium sulfate was used to remove water from extracted oil. Finally the oil was properly sealed in glass vials and stored at − 30 °C till further analysis/use in refrigerator (HF3-700S, USA).

### Gas chromatography–mass spectrometry (GC/MS) analysis

Essential oil of *E. umbellata* were analysed by means of an Agilent USB-393752 gas chromatograph (Agilent Technologies, Palo Alto, CA, USA). The instrument have the arrangement to effectively vaporizes the sample (the gas phase) and separates its various components using HP-5MS 5% phenyl methyl siloxane capillary column (30 m × 0.25 mm × 0.25 μm film thickness; Restek, Bellefonte, PA) and was equipped with an FID detector for phytoconstituents identification. The oven temperature was set first at 70 °C for 1 min, and then increased to 180 °C at the rate of 6 °C/min for 5 min and lastly to 280 °C for 20 min at the rate of 5 °C/min. Injector temperature was set at 220 °C while detector temperatures was set at 290 °C. The diluted samples (1/1000 in *n*-pentane, v/v) having volume 1 μL were injected manually in the split-less mode. Helium (49.6 psi) was used as carrier gas at a flow rate of 1 mL/min which propelled the compounds and also act as reagent gas that causes charge-exchange chemical ionization of the analytes which are then separated on the basis of mass-to-charge (*m/z*) ratios. These components are then identified through comparison with known standards in literature [20].

### Identification of components

The identification of major constituents of essential oil was done by comparison of their retention times and retention indices with those of authentic compounds reported in the literature. The essential oil components were identified by comparing their retention indices and mass spectral fragmentation patterns the compounds present with those reported in the Wiley and NIST libraries, mass spectral library, and also with mass spectral data reported in the literature [20, 21]. Kovats retention indices were also determined using formula:

1 $$ RIx=100n+100\left( tx- tn\right)/\left( tn+1- tn\right) $$

Where: RI*x* is the retention index of compound *x*, tn and tn + 1 are retention times of the reference n-alkane hydrocarbons eluting immediately before and after the compound *x*, and tx is the adjusted retention time of compound *x*.

### Antioxidant scavenging assays

#### DPPH free radicals assay

Brand-Williams assay [22] was used with some modification to check the scavenging potential of essential oil of *E. umbellata* against DPPH (2, 2-diphenyl-1-picrylhydrazyl) free radical. To prepare DPPH solution, 24 mg of it were dissolved in 100 mL methanol. Approximately, 1 mg/mL stock solution of essential oil was also prepared in methanol and serially diluted to obtain the dilution having concentrations; 1000, 500, 250, 125, 62.5 and 31.05 μg/mL. Subsequently 0.1 mL of each dilution was mixed with 3 mL of DPPH solution. The mixtures were incubated for 30 min at 25 °C. Absorbance was measured at wave length 517 nm through UV spectrophotometer (Thermo Electron Corporation; USA) and ascorbic acid was used as a positive control. All the samples were analysed in triplicates and the results are presented as Mean ± SEM. Percent DPPH scavenging potential was calculated using the following equation:

2 $$ Percent\ Scavenging\ potential=\frac{\boldsymbol{Blank}\ \boldsymbol{sample}\ \boldsymbol{absorbance}-\boldsymbol{sampleabsorbance}}{\boldsymbol{Blank}\ \boldsymbol{sample}\ \boldsymbol{absorbance}}\times 100 $$

#### ABTS free radical assay

The 2,2′-azinobis-3-ethylbenzothiazoline-6-sulfonic acid (ABTS) free radicals scavenging potential of essential oil was determined using the reported standard protocol [23, 24]. ABTS (7 mM) solution was thoroughly mixed with potassium persulfate (2.45 mM) solutions. The mixture was incubated overnight in dark for the production of free radicals. After that absorption of a 3 mL volume from it was adjusted at 745 nm to 0.7 through adding methanol (50%). About 300 μL of test samples were mixed with 3 mL ABTS solution and incubated for 6 min. The absorbance of mixtures were noted using UV spectrophotometer. Standard ascorbic acid was used as positive control. All the test samples were analysed in 3 replicates and percent ABTS scavenging potential was calculated using eq. 2.

#### In vitro anticholinesterase assays

Anticholinesterase inhibition potential of essential oil of *E. umbellata* were determined spectrophotometrically using the reported standard Ellman assay [25]. Acetyl choline iodide and butyrylcholine iodide were used as substrates. About 205 μL of essential oil having concentration in the range of 31.05–1000 μg/mL were added to a cuvette containing 5 μL of AChE (0.03 U/mL) and BChE (0.01 U/mL), through micropipette. Then 5 μL of DTNB was added to mixture kept in a water bath at 30 °C. After incubation for 15 min, 5 μL of substrates (acetylthiocholine iodide or butyrylthiocholine iodide) were added to the mixtures that resulted in yellow coloration (5-Thio-2-nitro benzoate anion color). Then the absorbance was recorded at 412 nm using double beam spectrophotometer (Thermo electron corporation, USA). A blank solution was prepared containing only essential oil but no enzyme. Galantamine was used as a positive control for which same procedure mentioned above was used. The absorption of each sample was recorded for 4 min. Percent enzyme activity and percent inhibition was calculated using the following equations:

3(a) $$ V=\Delta  Abs/\Delta  t. $$

3(b) $$ \% enzyme\ activity=\frac{V}{Vmax}\times 100. $$

3(c) $$ \% enzyme\ inhibition=100-\% enzyme\ activity. $$

Where: V is rate of reaction in presence of inhibitor while Vmax is the rate of reaction in absence of inhibitor.

#### In vitro α–glucosidase inhibition

The α-glucosidase inhibition potential of essential oil of *E. umbellata* were evaluated according to the reported assay [26] with some modifications. The reaction mixture was formulated by adding 100 μL of α-glucosidase (0.5 unit/mL), 600 μL of phosphate buffer (0.1 M; pH 6.9) and 50 μL of essential oil dilutions (31.05, 62.5, 125, 250, 500 and 1000 μg/mL). To initiate the reaction 100 μL *p*-nitro-phenyl-α-D-glucopyranoside (5 mM) solution was added into each reaction mixture. The resulting mixtures were incubated at 37 °C for 15 min. Then the reaction was stopped by the addition of 400 μL sodium carbonate (0.2 M) solution and the absorbance of mixture was recorded at 405 nm. Acarbose (2–100 μg/mL) was used as positive control. The reaction mixture with no essential oil was used as a negative control while the blank solution was prepared without α-glucosidase. The IC_50_ values of essential oil sample were calculated by plotting % α-glucosidase inhibition as a function of concentration. The % α-glucosidase inhibition potential was calculated using following equation.

4 $$ Percent\ enzyme\ Inhibition=\frac{\boldsymbol{control}\ \boldsymbol{absorbance}-\boldsymbol{sample}\ \boldsymbol{absorbance}}{\boldsymbol{control}\ \boldsymbol{absorbance}}X100 $$

#### In vitro α-amylase inhibition

The α-amylase inhibition potential was determined using 3,5-dinitrosalicylic acid (DNSA) assay [27]. About 1 mg/mL stock solution of essential oil of *E. umbellata* was dissolved in 10% DMSO, 0.02 M Na_2_HPO_4_/NaH_2_PO_4_ buffer and 0.006 M NaCl at pH 6.9. The stock solution of essential oil was serially diluted in the range of 31.05–1000 μg/mL. Then 200 μL of α-amylase (2 units/mL) solution was mixed with 200 μL essential oil and incubated at 30 °C for 10 min. Subsequently 200 μL starch (1% in water; w/v) solution was added to each dilution following incubation for 3 min. The reaction was stopped by adding 200 μL sodium potassium tartrate tetrahydrate dissolved in 8 mL NaOH (2 M) and 20 mL 3, 5 dinitrosalicylicacid (96 mM). The mixture was boiled for 10 min in a water bath at 85–90 °C. After cooling the mixture was diluted with 5 mL distilled water. The absorbance was recorded at 540 nm using UV-visible spectrophotometer. A blank solution was prepared containing only essential oil but no enzyme. Standard acarbose (2–100 μg/mL) was used as positive control. The same procedure mentioned above was used to prepare reaction mixture of positive control and absorbance was measured at 540 nm. The IC_50_ values of essential oil sample were calculated by plotting % α-amylase inhibition as a function of concentration. The α-amylase enzyme inhibition potential was calculated using the eq. 4.

### Statistical analysis

All the experiments were performed in three replicates. Two way ANOVA followed by Bonferroni Post-test (to determine the values of P) were applied to establish the statistical differences between standard drug and test samples using Graph Pad Prism software. The results were represented as Mean ± SEM. The results for which *P* < 0.05 were considered as significant. The medium inhibitory concentration (IC_50_) of DPPH, ABTS, AChE, BChE, α-glucosidase, and α-amylase enzyme were determined using linear regression analysis using MS Excel program 2007. R^2^ values that were used to establish correlation between the biological potentials (antioxidant and inhibition of AChE, BChE, α-glucosidase, and α-amylase) of essential oil samples and the respective standards used in the study, were calculated using Excel 2007.

## Results

### GC-MS results

**Fig. 1:**
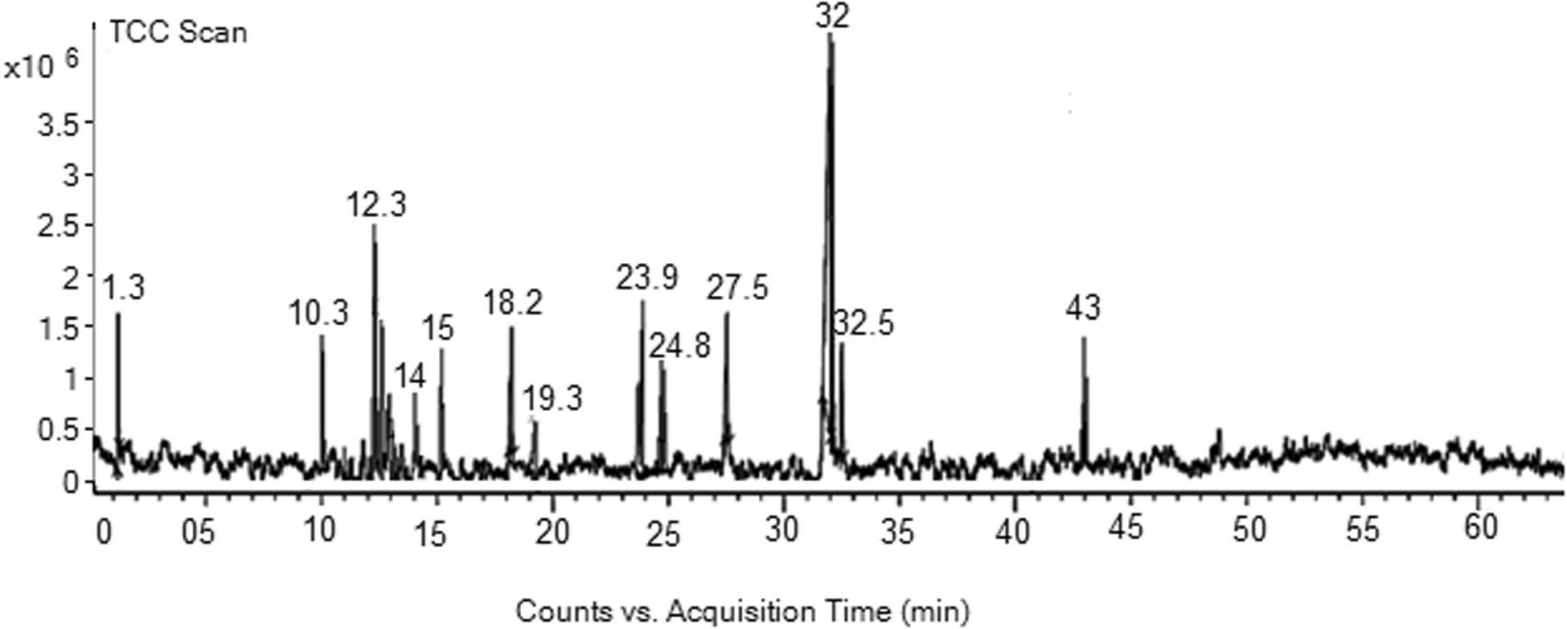
GC-MS chromatogram of the fruit essential oil of *Elaeagnus umbellata* Thunb

**Fig. 2:**
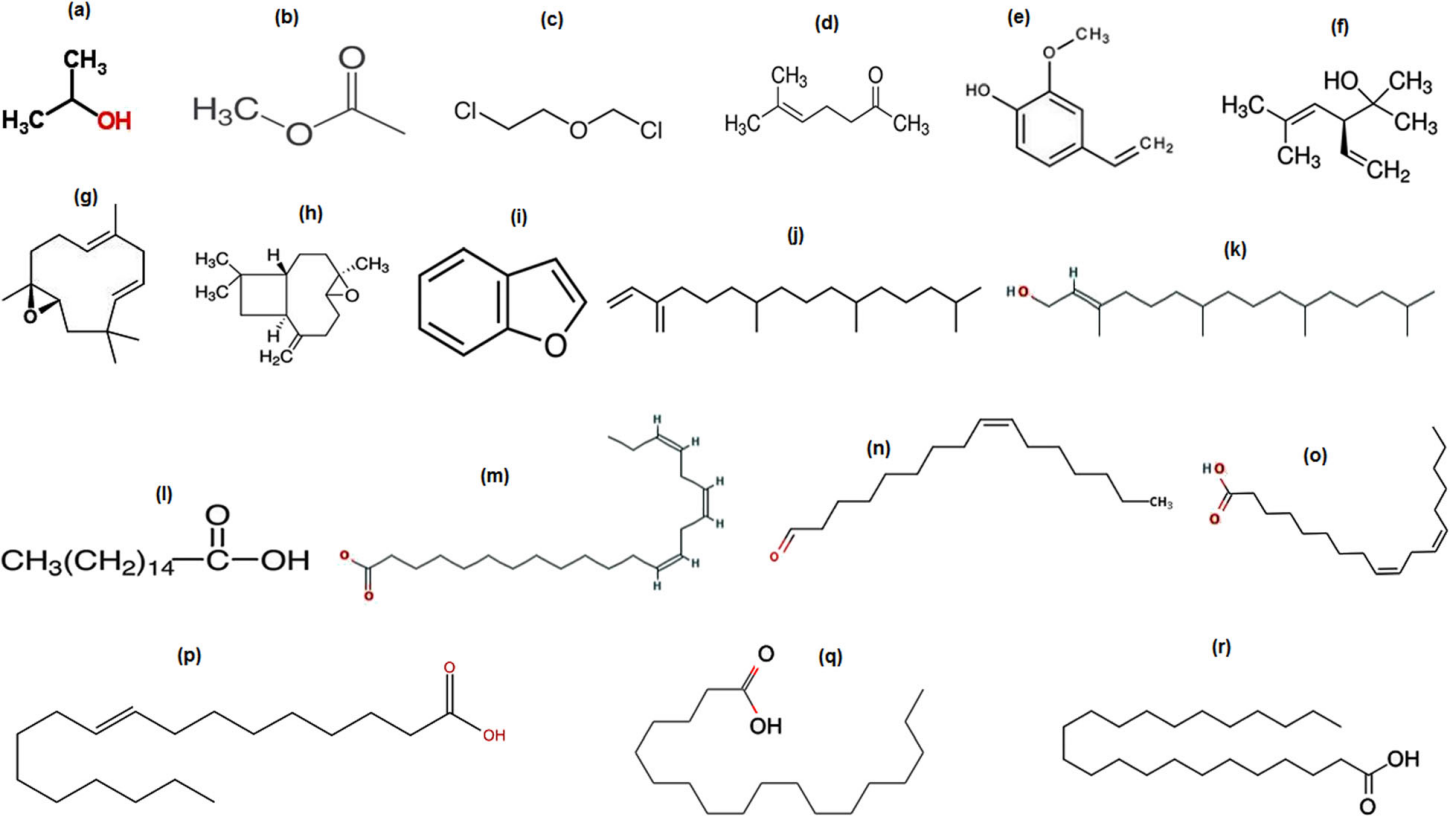
Major compounds identified in the fruits essential oil of *Elaeagnus umbellata* Thunb. via GC-MS [(**a**) Isopropyl alcohol, (**b**) Acetic acid, methyl ester, (**c**) Bis -dichloromethyl -ether, (**d**) 5-Hepten-2-one, 6-methyl, (**e**) 2-Methoxy-4-vinylphenol (*p*-Vinylguaiacol), (**f**) 2,5-Dimethyl-3-vinyl-4-hexen-2ol (α-santolina alcohol), (**g**) Humulene Oxide, (**h**) (−)Caryophyllene oxide, (**i**) Benzofuran, (**j**) Neophytadiene, (**k**) 3,7,11,15-Tetramethyl-2-hexadecen-1-ol, (**l**) n-hexadecanoic acid, (**m**) 8, 11, 14- Docosatrienoic acid, (**n**) *cis*-9-Hexadecenal, (**o**) *Cis-cis*-9,12-Octadecadienoic acid (Linoleic acid), (**p**) 9-Octadecenoic acid, (**e**)-, (**q**) Octadecanoic acid, (**r**) Tricosanoic acid]

The GC-MS chromatogram of essential oil of *E. umbellata* is shown in Fig. 1. A total of 68 compounds were identified among the detected compounds in the essential oil sample. Some of these compounds are previously reported to have antioxidant and anticholinesterase potentials [15, 17, 28]. Out of the identified compounds as shown in table the major constituents are: Isopropyl alcohol, acetic acid, methyl ester, bis-dichloromethyl–ether, 5-hepten-2-one, 6-methyl, 2-methoxy-4-vinylphenol, 2,5-dimethyl-3-vinyl-4-hexen-2 ol, humulene Oxide, (−)caryophyllene oxide, benzofuran, neophytadiene, 3,7,11,15-tetramethyl-2-hexadecen-1-ol, n-hexadecanoic acid, 8, 11, 14- docosatrienoic acid, cis-9-hexadecenal, Cis-cis-9, 12-octadecadienoic acid, 9-Octadecenoic acid, (E)-, octadecanoic acid, and tricosanoic acid that were eluted from GC column at retention times; 1.39,1.43, 1.60, 12.71, 12.05, 15.02, 18.80, 19.21, 19.32, 24.05, 24.83, 27.55, 31.00, 32.01, 32.08, 32.21, 32.59, and 43.03 min respectively. Among these compounds the most active components reported in literature are presented in Table S1 (Supplementary file) while their chemical structures are given in Fig. 2.

### Antioxidant activities

**Fig. 3:**
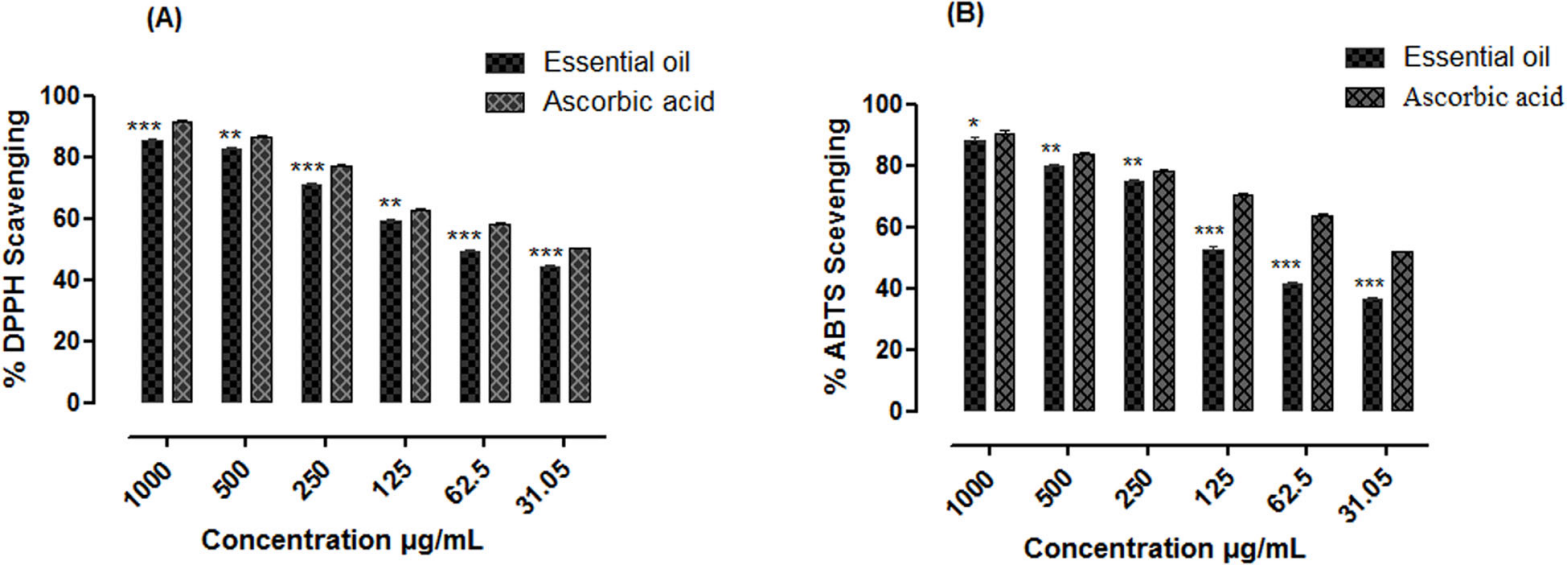
Antioxidant potential of the essential oil from the fruits of *Elaeagnus umbellata* Thunb. [(**a**) DPPH and (**b**) ABTS free radical scavenging activity of essential oil of *Elaeagnus umbellata*. The data is represented as Mean ± SEM, *n* = 3. (*, ** and *** indicates that values were significantly different (*P*< 0.05, *P*< 0.01, *P*< 0.001) as compared to positive control. Significance difference (*P< 0.05, **p < 0.01 and ***p < 0.001) were made between the test samples (Essential oil) versus positive control (ascorbic acid) by Two way ANOVA followed by Bonferroni Post-test]

The observed free radicals scavenging potential of essential oil sample of *E. umbellata* estimated through DPPH and ABTS assays, was significant and comparable with that of the positive control ascorbic acid. The highest percent scavenging potential observed were; 85.24 ± 0.63, 88.30 ± 0.81 respectively against DPPH (Fig. 3a) and ABTS (Fig. 3b) at the highest concentration of 1000 μg/mL with IC_50_ values of 70 and 105 μg/mL respectively. The standard ascorbic acid caused an inhibition of 91.56 ± 0.35 and 92.63 ± 0.99% against DPPH and ABTS at the highest concentration 1000 μg/mL with IC_50_ values of 32 and 29 μg/mL respectively.

### Anticholinesterase activities

**Fig. 4:**
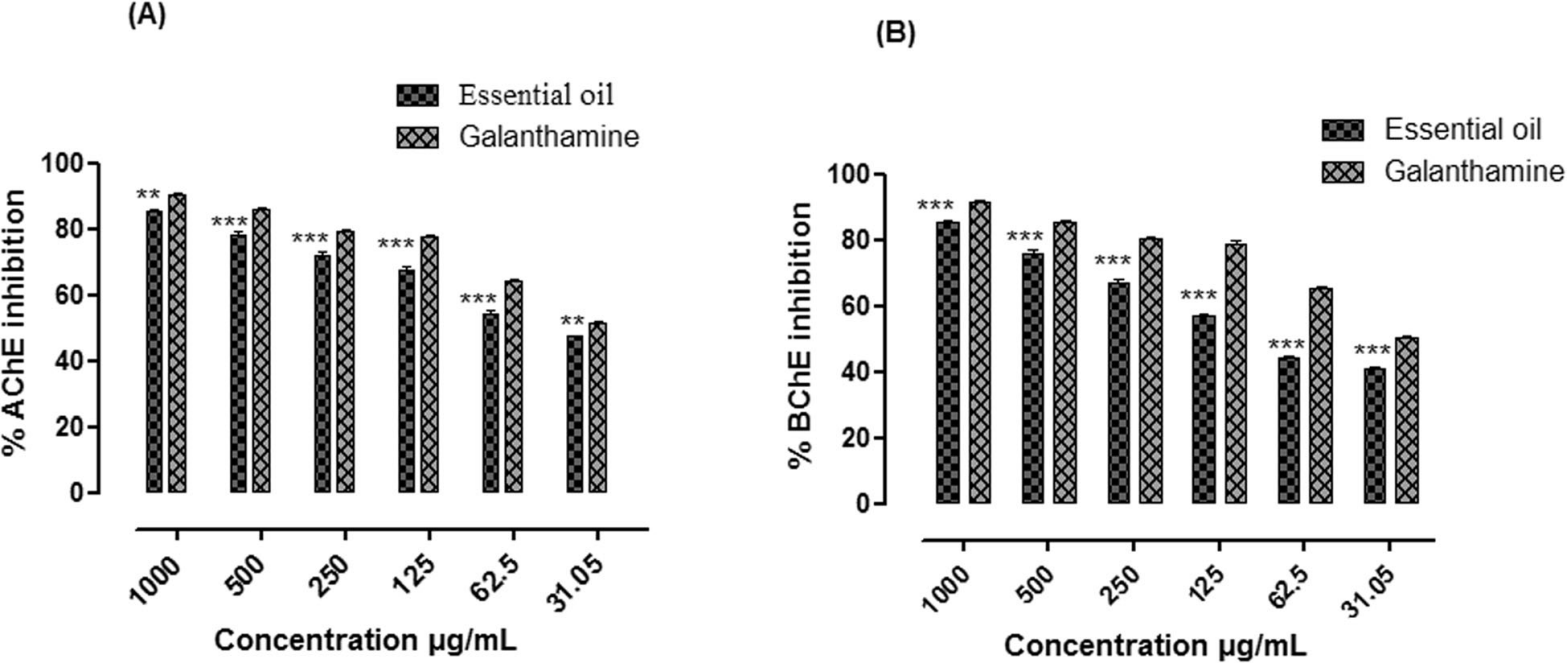
Anticholinesterase potential of the essential oil from the fruits of *Elaeagnus umbellata* Thunb. [(**a**) %AChE and (**b**) %BChE inhibition potential of essential oil of *Elaeagnus umbellata*. The data is represented as Mean ± SEM, *n* = 3. (** and *** indicates that values were significantly different (*P*< 0.01, *P*< 0.001) as compared to positive control. Significance difference (**p < 0.01 and ***p < 0.001) were made between the test samples (Essential oil) versus positive control (galantamine) by Two way ANOVA followed by Bonferroni Post-test]

The anticholinesterase potential of essential oil has been summarized in the Table S2 (Supplementary file). The observed anticholinesterase potential is probably due to the presence of active chemical constituents (Table 1). Essential oil demonstrated 85.44, 78.07, 71.86 67.59, 54.37, and 47.37% AChE inhibition at 1000, 500, 250, 125, 62.5, and 31.05 μg/mL concentrations respectively with IC_50_ = 48 μg/mL (Fig. 4a). Similarly, the %BChE inhibition recorded were; 81.45, 76.08, 67.13, 56.82, 44.11, and 40.66 at 1000, 500, 250, 125, and 62.5 μg/mL concentrations respectively with IC_50_ = 90 μg/mL (Fig. 4b). The anticholinesterase potential of essential oil was comparable with the positive control galantamine (AChE IC_50_ = 25 μg/mL) and (BChE IC_50_ = 30 μg/mL).

**Table 1:** Chemical composition of the essential oil from the fruits of *E. umbellata* Thunb. via GC-MS

S.No	RT	RI	Mol. wt	Mol. formula	Compound name	Content (%)	Hits (DB)
1	1.31	484	60	C_2_H_4_O_2_	Methyl formate	0.1	5
2	1.32	651	44	C_2_H_4_O	Acetaldehyde	0.8	5
3	1.33	282	60	C_3_H_8_O	Isopropyl alcohol	5.4	5
4	1.38	671	86	C_5_H_10_O	4-Penten-2-ol	1.2	5
5	1.39	681	88	C_5_H_10_O	2- Pentanol	1.0	5
6	1.40	515	74	C_3_H_6_O_2_	Methyl ethanoate	0.4	5
7	1.41	722	102	C_4_H_6_O_3_	Propanoic acid	0.6	4
8	1.42	487	74	C_3_H_6_O_2_	Acetic acid, methyl ester	6.4	5
9	1.43	576	60	C2H4O2	Acetic acid	1.2	5
10	1.43	850	130	C_7_H_14_O_2_	Butanoic acid, 2-methyl-, ethyl ester	0.2	5
11	1.59	699	182	C_2_H_2_Cl_4_O	Bis -dichloromethyl -ether	8.5	3
12	1.60	662	74	C_4_H_10_O	1-Butanol	1.4	5
13	1.68	783	102	C_5_H_10_O_2_	*n*-Butyl-formate	0.4	4
14	1.69	913	132	C_6_H_12_OS	Ethanethioic acid, S- (2-methylpropyl) ester	0.9	5
15	6.66	1074	120	C_4_H_8_O_4_	1,4-Dioxane-2,5-diol	0.7	5
16	7.47	1068	116	C_5_H_8_O_3_	Pentanoic acid, 4-oxo	9.5	1
17	10.01	1185	156	C_10_H_20_O	Decanal	0.7	5
18	10.32	1732	176	C_10_H_8_O_3_	Coumarin, 7-methoxy	15.4	5
19	11.41	1056	130	C_8_H_18_O	5-methyl −3-Heptanol	0.5	5
20	12.05	1504	212	C_13_H_24_O_2_	*Cis*-5-Dodecenoic acid, methyl ester	13.1	4
21	12.31	1316	150	C_9_H_10_O_2_	2-Methoxy-4-vinylphenol, (p-Vinylguaiacol)	10.5	5
22	12.71	987	128	C_8_H_16_O	5-Hepten-2-one, 6-methyl	12.4	4
23	13.59	1374	172	C_10_H_20_O_2_	*n*-Decanoic acid	11.3	5
24	14.23	878	144	C_8_H_16_O_2_	Ethyl 3,3-dimethylbutanoate	0.4	5
25	14.77	934	116	C_5_H_8_O_3_	4-hydroxy, 2-Pentenoic acid	1.2	10
26	15.02	1038	154	C_10_H_18_O	2,5-Dimethyl-3-vinyl-4-hexen-2-ol	9.8	10
27	15.05	1436	186	C_10_H_18_O_3_	Nonanoic acid, 9-oxo-methyl ester	1.3	10
28	18.01	1307	158	C_9_H_18_O_2_	Nonanoic acid	1.9	10
29	18.21	1421	204	C_15_H_24_	(−)-Caryophyllene	31.2	5
30	18.80	1600	220	C_15_H_24_O	Humulene Epoxide	25.4	5
31	19.31	1570	220	C_15_H_24_O	(−) Caryophyllene oxide	39.4	5
32	19.31	1561	255	C_16_H_17_NO_2_	1-Phenyl-2-(4-methylphenyl)-3-nitropropane	1.4	5
33	19.32	1005	118	C_8_H_6_O_2_	Benzofuran	9.5	5
34	20.51	1522	218	C_9_H_9_NO_4_	2,3-Pyridinedicarboxylic acid, dimethyl ester	0.5	5
35	22.17	1692	222	C_14_H_22_O_2_	2,2,6-Trimethyl-1-(3-methylbuta-1,3-dienyl)-7-oxabicyclo [4.1.0]heptan-3-ol	1.5	4
36	23.91	1723	222	C_15_H_26_O	3,3,7-Trimethyltricyclo [5.3.1.02.8] undecane-11-methanol, (−)-Isolongifolol	3.5	4
37	24.05	1806	278	C_20_H_38_	7,11,15-trimethyl-3-methylidenehexadec-1-ene (Neophytadiene)	11.3	10
38	24.82	1869	242	C_15_H_30_O_2_	Pentadecanoic acid	4.0	10
39	24.83	2114	296	C_20_H_40_O	3,7,11,15-Tetramethyl-2-hexadecen-1-ol (Phytol)	10.4	10
40	24.90	2101	292	C_19_H_32_O_2_	9,12,15-Octadecatrienoic acid, methyl ester	4.1	10
41	25.03	2114	280	C_20_H_40_O	3,7,11,15-Tetramethyl-2-hexadecen-1-ol	2.5	5
42	25.03	3378	469	C_32_H_52_O_2_	9,19-Cycloergost-24, 28-en-3-ol, 4,14-dimethyl acetate	1.1	5
43	25.30	1195	172	C_10_H_20_O_2_	Octanoic acid, ethyl ester	0.9	5
44	27.55	1968	256	C_16_H_32_O_2_	*n*-hexadecanoic acid (Palmitic acid)	0.4	5
45	27.70	4765	653	C_38_H_68_O_8_	(L-Ascorbyl 2,6-Dipalmitate) (+)-Ascorbic acid 2,6-dihexadecanoate	15.4	5
46	27.75	1869	242	C_15_H_30_O_2_	Pentadecanoic acid	4.2	5
47	30.85	2183	280	C_18_H_32_O_2_	*Cis-cis*-9,12- octadecadienoic acid (Linoleic acid)	1.5	4
48	30.85	2721	352	C_21_H_36_O_4_	Linolenic acid, 2-hydroxy-1-(hydroxymethyl) ethyl ester	7.9	4
49	31.00	2499	348	C_23_H_40_O_2_	8, 11, 14- Docosatrienoic acid	11.0	5
50	32.01	2300	320	C_20_H_34_O_2_	8, 11, 14-Eicosatrienoic acid	0.5	5
51	31.05	2101	292	C_18_H_30_O2	9, 12, 15-Octadecatrienoic acid	8.8	5
52	31.07	2266	334	C_11_H_20_O_2_	Cyclo propane octanoic acid	1.5	5
53	32.08	2093	294	C_18_H_32_O_2_	*Cis-cis*-9, 12-Octadecadienoic acid-methyl ester	14.0	5
54	32.03	2808	238	C_16_H_30_O	*Cis*-9-Hexadecanal	9.0	1
55	32.07	1609	210	C_14_H_26_O	7-Tetradecanal	2.5	3
56	32.08	2042	306	C_15_H_24_O_2_	Dichloroacetic acid, tridec-2-ynyl ester	3.6	4
57	32.09	2007	266	C_18_H_34_O	9-Octadecenal	4.7	5
58	32.10	2292	322	C_21_H_38_O_2_	*Cis*-11,14-Eicosadienoic acid, methyl ester	1.8	5
59	32.21	2175	282	C_18_H_34_O_2_	9-Octadecenoic acid	9.4	4
60	32.23	2230	296	C_19_H_36_O_2_	10, 2-Hexacyclo propyl decanoic acid	2.5	4
61	32.59	2167	284	C_18_H_36_O_2_	Octadecanoic acid	14.1	1
62	32.60	2266	334	C_22_H_38_O_2_	Cyclopropaneoctanoic acid	2.7	3
63	32.72	1226	138	C_8_H_14_N_2_	1-Butyl-2-methyl-1H-imidazole	1.5	3
64	43.00	1897	250	C_9_H_18_N_2_O_4_S	2-Butanone, 3,3-dimethyl-1-(methylsulfonyl)-, O-[(methylamino)carbonyl]oxime	1.4	5
65	43.03	2715	424	C_28_H_56_O_2_	Tricosanoic acid	19.5	5
66	43.05	2600	320	C_16_H_17_ClN_2_O_3_	4 (4-Chlorophenyl)-3 morpholinopyrrol-2-carboxylic acid	0.1	5
67	43.07	1034	172	C_10_H_20_O_2_	2-t-Butylpentanoic acid	1.3	5
68	44.34	2241	294	C_19_H_34_O_2_	E, E, Z-1, 3, 12-Nonadecatriene-5, 14-diol	0.7	2

*RT* Retention time, *RI* Retention indices

### In vitro α-glucosidase and α-amylase inhibition

**Fig. 5:**
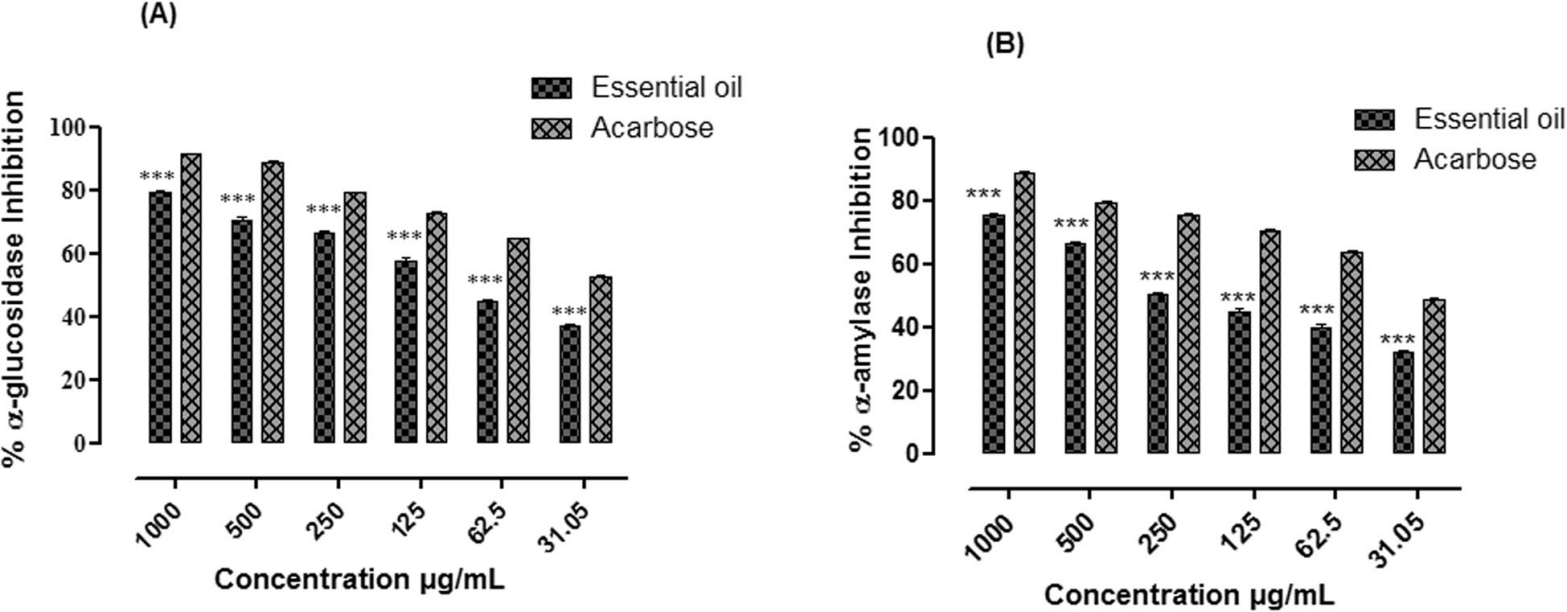
Antidiabetic potential of the essential oil from the fruits of *Elaeagnus umbellata* Thunb. [(**a**) % α- glucosidase inhibition and (**b**) % α-amylase inhibition potential of essential oil of *Elaeagnus umbellata*. The data is represented as Mean ± SEM, *n* = 3. (*** indicates that values were significantly different (*P*< 0.001) as compared to positive control. Significance difference (***p < 0.001) were made between the test samples (Essential oil) versus positive control (acarbose) by Two way ANOVA followed by Bonferroni Post-test]

The percent α-glucosidase and α-amylase inhibition potential of essential oil of *E. umbellata* are presented in Table S3 (Supplementary file). The % α-glucosidase inhibition potential of essential oil sample observed were; 75.25, 69.61, 60.56, 52.51, 32.74, and 30.61 at 1000, 500, 250, 125, 62.5, and 31.05 μg/mL concentrations respectively with IC_50_ value of 120 μg/mL (Fig. 5a). The %α-amylase enzyme inhibition potentials were; 88.30, 79.85, 74.82, 52.51, 41.39, and 36.24 at 1000, 500, 250, 125, 62.5, 31.05 μg/mL concentrations with IC_50_ of 110 μg/mL respectively (Fig. 5b). Acarbose a standard inhibitor of α-glucosidase and α-amylase produced an IC_50_ values of 28 and 30 μg/mL respectively against the selected enzymes.

### Linear correlation between *E. umbellata* essential oil sample vs antioxidants, anticholinesterase, and antidiabetic activities

**Fig. 6:**
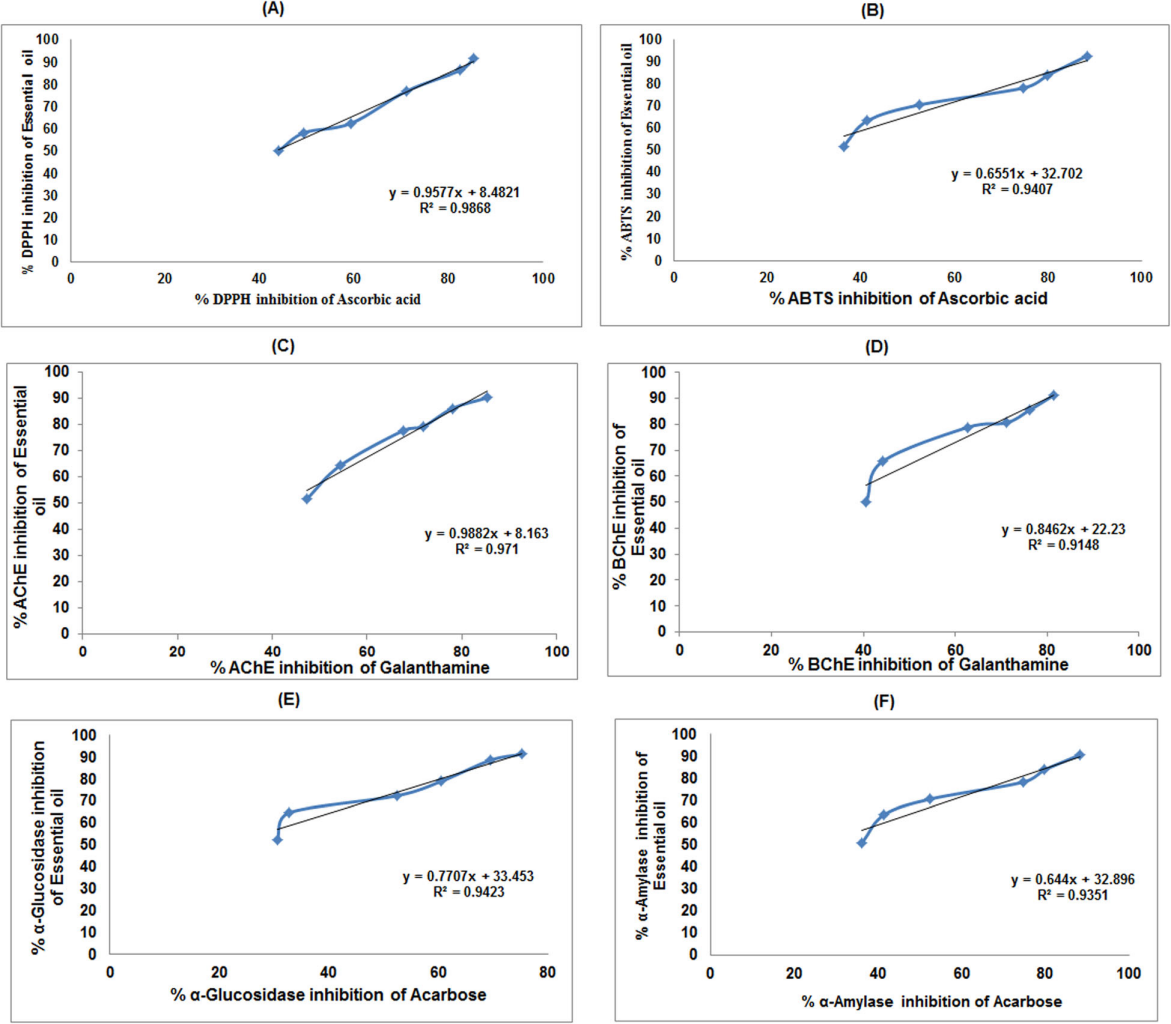
Antioxidant, anticholinesterase, and antidiabetic potential of the essential oil from the fruits of *Elaeagnus umbellata* Thunb. and their linear correlation with standard ascorbic acid, galantamine, and acarbose [(**a**) Percent DPPH and (**b**) ABTS free radical scavenging activity, (**c**) Percent acetyl cholinesterase (AChE), and (**d**) buterly cholinesterase (BChE) inhibition, (**e**) Percent α-glucosidase inhibition, and (**f**) Percent α-amylase inhibition potential of essential oil of *Elaeagnus umbellata*]

A linear correlation between the observed biological activities of *E. umbellata* essential oil sample vs observed activities exhibited by the standard used (Ascorbic acid, galantamine, and acarbose) have been presented in Fig. 6. The regression value for % DPPH inhibition by essential oil and ascorbic acid (Fig. 6a) is 0.9868 while against ABTS (Fig. 6b) it is 0.9407.

Similarly, the regression value of %AChE inhibition by essential oil sample and standard is 0.971 (Fig. 6c) while in case of BChE (Fig. 6d) it is 0.9148. The acetyl cholinesterase and butyryl cholinesterase inhibition potential shown by essential oil sample of *E. umbellata* were comparable with that of positive control galantamine which is also obvious from the correlation coefficient values. The regression value for % α-glucosidase and α-amylase inhibition by essential oil sample (Fig. 6e, f) vs standard acarbose were 0.9423 and 0.9351 respectively. From the regression values it was concluded that essential oil have comparable antioxidant, anticholinesterase, and antidiabetic capabilities in comparison to used standards.

## Discussions

In this study essential oils were extracted from *E. umbellata* which were then fractionated through GCMS and after comparison of their retention times with those reported in literature, 68 compounds were identified. The essential oil of *E. umbellata* were then evaluated for their antioxidant, antidiabetic and anticholinesterase potentials using standard assays and substantial activities were recorded.

Plant and their products are used by human as important health remedies since time immemorial. Plants are considered to be the natural product factories as they are self-nourished that also have the capabilities to cope with uneven situations. In uneven situation they produce certain chemical called secondary metabolites which are used as tool of offence and defence. Most of these metabolites contain phenolic rings and are natural antioxidants. There is need to isolate them in pure state which would leads to the development of novel drugs [29]. Natural antioxidants are potentially safe as they have limited side effects, efficient in term of their efficacies and inexpensive as they are obtained from renewable sources. Epidemiologically a relation has been established between plant antioxidants and reduction in a number of certain chronic disorders [30]. Literature studies have demonstrated that dietary antioxidants obtained from fruits and vegetables can effectively scavenge the free radicals formed during metabolism [31].

**Fig. 7:**
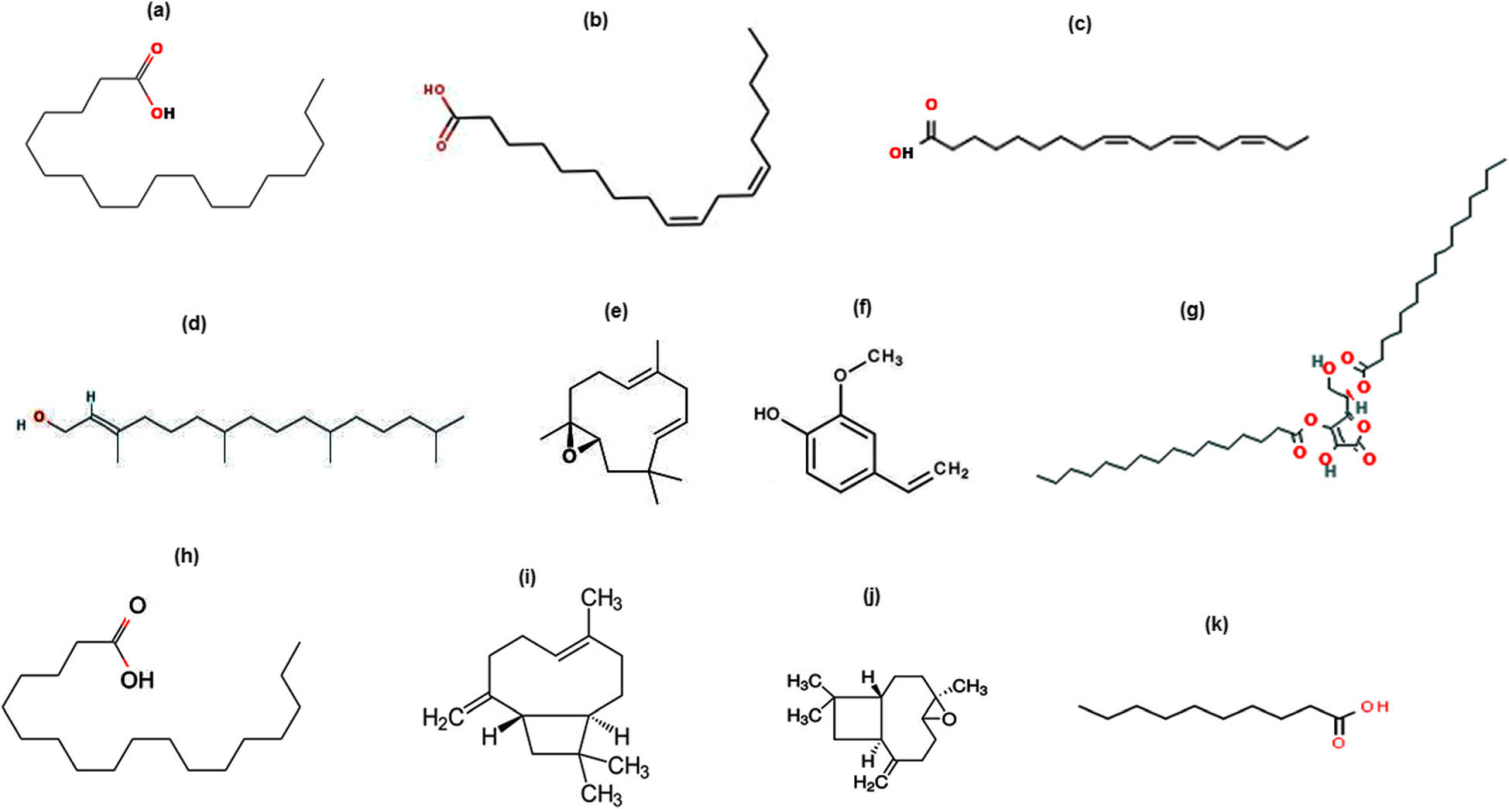
Active antioxidant, antidiabetic, and neuroprotective compounds identified in the fruits essential oil of *Elaeagnus umbellata* Thunb. via GC-MS [(**a**) Octadecanoic acid, (**b**) *Cis-cis*-9,12-Octadecadienoic acid (Linoleic acid), (**c**) α-linolenic, (**d**) 3,7,11,15-Tetramethyl-2-hexadecen-1-ol (Phytol), (**e**) Humulene Epoxide, (**f**) 2-Methoxy-4-vinylphenol (*p*-Vinylguaiacol), (**g**) (+)-Ascorbic acid 2,6-dihexadecanoate, (**h**) Octadecanoic acid (Steric acid) (**i**) Caryophyllene, (**j**) (−)Caryophyllene oxide, (**k**) Decanoic acid]

Natural antioxidants, usually belongs to phenolic and flavonoid categories of phytochemicals. However, it should be noted that flavonoids are larger compounds and are usually not present in essential oils. At the same time phenolic are also very few in them. There are many volatile components in such samples responsible for the antioxidant activities (described below). *E. umbellata* is the least explored plant. Although antibacterial, anti-fungal, insecticidal, phytotoxic activities, free radical scavenging, antidiabetic, and anti-amnesic activity has been reported [13, 14, 32, 33], but essential oil of fruit has not been investigated yet. Substantial DPPH and ABTS free radical scavenging activities were observed for the extracted oil which may be due to the chemical constituents as indicated in GC-MS results (Fig. 7). The octadecanoic acid has been reported with significant antioxidant potential [34]. Similarly, the *cis-cis*-9,12-Octadecadienoic acid which is commonly known as linoleic acid is an antioxidant compound [35]. Likewise the α-linolenic acid [36], and 3,7,11,15-tetramethyl-2-hexadecen-1-ol (Phytol) reportedly possess free radicals scavenging potential [37, 38]. Humulene epoxide is also a moderate antioxidant [39]. The 2-Mwthoxy-4-vinylphenol i.e., p-Vinylguaiacol is a significant radical scavenger [40, 41]. As far as the (+)-ascorbic acid 2,6- dihexadecanoate is concerned, this is used as a reference standard in free radicals scavenging activities [42, 43].

The inhibitors of carbohydrate digesting enzymes, α-glucosidase and α-amylase prevent the absorption of dietary starch and decrease the postprandial glucose level. Efficient inhibitors are needed to inhibit them in diabetes mellitus to keep in control the blood glucose level. The observed antidiabetic potential of *E. umbellata* fruit essential oil may be due to the presence of important constituents as indicated in the Fig. 7. The antidiabetic activities of α-linolenic acid (identified in the GC-MS analysis of *E. umbellata*), has been previously reported [44, 45]. The 3,7,11,15-tetramethyl-2-hexadecen-1-ol which is also known as phytol have been reported to have strong antidiabetic potential [46]. Similarly, the octadecanoic acid or steric acid also possess antidiabetic potential [47]. The humulene epoxide also have the capacity to lower the blood glucose level [39], and the (+)-ascorbic acid 2,6-dihexadecanoate is one of the component of the essential oil of *E. umbellata*, which has been reported to possess antidiabetic potential [48]. The essential oil inhibited α-glucosidase and α-amylase efficiently and the results were comparable with acarbose a standard inhibitor of these enzymes that inhibited both of them with IC_50_ values of 28 and 30 μg/mL respectively.

Excellent inhibition of AChE and BChE by essential oil were observed in this study. Thus it is suggested that these oil could be used as alternative drug to treat neurological disorders. They would probably enhance the cholinergic transmission, reduction of beta amyloid aggregation, and formation of the neurotoxic fibrils in Alzheimer’s disease [49]. Our results are comparable with the previously reported data on *E. umbellata* that verifies its antioxidant and anticholinesterase potentials [50, 51]. Essential oils obtained from various medicinal plants possess noticeable antioxidant and anticholinesterase potentials due to presence of a variety of valuable compounds present [52–54].

Our previously reported data on *E. umbellata* verifies its antioxidant, antidiabetic, anticholinesterase, and neuroprotective potentials [13, 14]. Some of the compounds present in the essential oil of *E. umbellata* (Fig. 7) have already been confirmed to possess neuroprotective activities for instance, the *cis-cis*-9,12-Octadecadienoic acid (Linoleic acid) [55]. In the same manner α-linolenic acid [56], caryophyllene [57], octadecanoic acid (stearic acid) [58], and decanoic acid have been recently reported to have strong neuroprotective activities [59]. Although a number of chemical compounds are present in the essential oil of *E. umbellata*, the most active compounds responsible for the observed biological activities were nominated based on reported studies in literature [55–59].

In support of the findings of the study linear regression coefficients were calculated. Regression coefficient values near to 1 were observed for almost all the three biological activities performed indicating that these oil have antioxidant, anticholinesterase, and antidiabetic activities which is also obvious from the reported studies [60, 61].

The study was limited to in vitro biological evaluation of the essential oil therefore the bioavailability and toxicological aspects have not been studied. In our future study these aspects will also be evaluated.

## Conclusion

In this study, the chemical composition of the essential oils extracted from *E. umbellata* fruit was determined. Out of the detected compounds (through GC-MS analysis), 68 were identified. As a rich source of valuable phytochemicals the extracted oils demonstrated antioxidant, anticholinesterase, and antidiabetic activities. The essential oil of this plant could therefore be used as alternative drug to treat oxidative stress related diseases. However, further studies are needed to identify the responsible compounds and test them individually for the observed biological potentials in in vitro and in vivo. Toxicological aspects and bioavailability evaluations of them are also important and needs to be evaluated.

## Supplementary Information

**Additional file 1: Table S1.** Phytochemical composition of essential oil extracted from *E. umbellata* Thunb. Fruit (determined through GC-MS). **Table S2.** Percent anticholinesterase (AChE and BChE) inhibition potential of the essential oil of *E. umbellata* fruit. **Table S3.** Percent α-glucosidase and α-amylase inhibition potential of the essential oil of *E. umbellata* fruit.

## Data Availability

The data presented in this manuscript belong to the PhD work of Dr. Nausheen Nazir and has not been deposited in any repository yet. However, the materials are available to the researchers upon request.

## Abbreviations

AD: Alzheimer’s Disease
Ach: Acetylcholine
AChE: Acetylcholine esterase
BChE: Butyrylcholine esterase
GC: Gas Chromatography
GC-MS: Gas chromatography-mass spectrometry
TPC: Total phenolic content
DPPH: 2, 20-diphenyl-1-picrylhydrazyl
ABTS: 2, 2’-azinobis-3-ethylbenzothiazoline-6-sulfonic acid
DTNB: 5,5-dithio-bis-2-nitrobenzoic acid
DNSA: 3, 5-dinitrosalicylic acid
Rt: Retention time
SEM: Standard error mean
IC_50_: Median inhibitory concentration
ROS: Reactive oxygen species
