# Supplementary material for: Chemical composition, in vitro antioxidant, anticholinesterase, and antidiabetic potential of essential oil of Elaeagnus umbellata Thunb
Source: BMC Complement Med Ther. 2021 Feb 22;21:73. doi: 10.1186/s12906-021-03228-y (PMC7898454; doi:10.1186/s12906-021-03228-y)
Supplement: Supplementary file 1 — Additional file 1: Table S1. Phytochemical composition of essential oil extracted from E. umbellata Thunb. Fruit (determined through GC-MS). Table S2. Percent anticholinesterase (AChE and BChE) inhibition potential of the essential oil of E. umbellata fruit. Table S3. Percent α-glucosidase and α-amylase inhibition potential of the essential oil of E. umbellata fruit. [file 12906_2021_3228_MOESM1_ESM.docx]

**Supplementary material**

**Tables**

**Table S1:** Phytochemical composition of essential oil extracted from *E. umbellata* Thunb. Fruit (determined through GC-MS)

| **S.No** | **RT** | **RI** | **Area** | **Area %** | **Height** | **Height %** | **Area Sum %** | **Base Peak m/z** | **Mass peak** | **Compound name** |
| --- | --- | --- | --- | --- | --- | --- | --- | --- | --- | --- |
| 1 | 1.39 | 282 | 22509 | 0.63 | 17454 | 0.76 | 1.29 | 43.10 | 455 | Isopropyl alcohol |
| 2 | 1.43 | 487 | 43059 | 1.41 | 32253 | 1.72 | 1.34 | 82.95 | 517 | Acetic acid, methyl ester |
| 3 | 1.60 | 699 | 286810 | 8.01 | 282261 | 12.37 | 1.02 | 83.05 | 515 | Bis -dichloromethyl -ether |
| 4 | 12.71 | 987 | 11586984 | 100 | 14335131 | 100 | 33.04 | 120 | 560 | 5-Hepten-2-one, 6-methyl |
| 5 | 12.05 | 1316 | 70226678 | 48.69 | 66352765 | 46.85 | 17.04 | 150 | 555 | 2-Methoxy-4-vinylphenol |
| 6 | 15.02 | 1038 | 5640019 | 38.99 | 55814679 | 55.51 | 13.35 | 94.91 | 512 | 2,5-Dimethyl-3-vinyl-4-hexen-2 ol |
| 7 | 18.80 | 1600 | 792137 | 5.71 | 582813 | 23.51 | 3.64 | 42.92 | 445 | Humulene Oxide |
| 8 | 19.21 | 1570 | 2284319 | 14.71 | 570779 | 23.25 | 4.22 | 67.01 | 480 | (-)Caryophyllene oxide |
| 9 | 19.32 | 1005 | 2788109 | 13.41 | 546134 | 23.09 | 3.09 | 202.91 | 620 | Benzofuran |
| 10 | 24.05 | 1806 | 9961820 | 100 | 118755 | 100 | 25.02 | 68.01 | 422 | Neophytadiene |
| 11 | 24.83 | 2114 | 8504473 | 25.46 | 625592 | 25.98 | 6.46 | 81.12 | 516 | 3,7,11,15-Tetramethyl-2-hexadecen-1-ol |
| 12 | 27.55 | 1968 | 442310 | 8.45 | 85312 | 10.67 | 5.18 | 73.05 | 503 | *n*-hexadecanoic acid |
| 13 | 31.00 | 2499 | 186638 | 4.09 | 35911 | 1.34 | 5.20 | 79.10 | 458 | 8, 11, 14- Docosatrienoic acid |
| 14 | 32.01 | 2808 | 108272 | 2.37 | 36977 | 1.38 | 2.93 | 55.10 | 475 | *cis*-9-Hexadecenal |
| 15 | 32.08 | 2093 | 2914832 | 55.66 | 260362 | 32.57 | 11.20 | 67.10 | 464 | *Cis-cis*-9, 12-Octadecadienoic acid |
| 16 | 32.21 | 2175 | 1451139 | 27.71 | 278746 | 34.87 | 5.21 | 55.05 | 511 | 9-Octadecenoic acid, (E)- |
| 17 | 32.59 | 2167 | 272379 | 5.20 | 78422 | 9.81 | 3.47 | 43.10 | 530 | Octadecanoic acid |
| 18 | 43.03 | 2715 | 95010 | 2.56 | 12959 | 0.53 | 7.33 | 18.30 | 548 | Tricosanoic acid |

Retention time, RT; Retention indices, RI

Table S2: Percent anticholinesterase (AChE and BChE) inhibition potential of the essential oil of *E. umbellata* fruit.

| **S.No** | **Sample** | **Concentration**  **(µg/mL)** | **% AChE** | **AChE IC_50_**  **(µg/mL)** | **% BChE** | **BChE IC_50_**  **(µg/mL)** |
| --- | --- | --- | --- | --- | --- | --- |
|  |  |  | **Mean ± SEM** |  | **Mean ± SEM** |  |
| 1 | Essential oil | 1000  500  250  125  62.5  31.05 | 85.44 ± 0.35**  78.07 ± 1.20***  71.86 ± 1.29***  67.59 ± 1.22***  54.37 ± 0.90***  47.37 ± 0.32** | 48 | 81.45 ± 0.68***  76.08 ± 0.71***  71.13 ± 1.04***  62.82 ± 0.98***  44.11 ± 0.64***  40.66 ± 0.72*** | 90 |
| 2 | Standard  Galantamine | 1000  500  250  125  62.5  31.05 | 90.16 ± 0.67  86.11 ± 0.23  79.12 ± 0.58  77.55 ± 0.51  64.29 ± 0.55  51.66 ± 0.49 | 25 | 91.22 ± 0.61  85.36 ± 0.48  80.56 ± 0.33  78.89 ± 0.65  65.58 ± 0.33  50.16 ± 0.56 | 30 |

AChE, Acetyl cholinesterase; BChE, Butyrylcholinesterase; The data is represented as mean ± SEM, (n = 3). Values are significantly different as compare to positive control Galantamine (**p < 0.01 and ***p < 0.001).

**Table S3:** Percent α-glucosidase and α-amylase inhibition potential of the essential oil of *E. umbellata* fruit.

| **S.No** | **Sample** | **Concentration**  **(µg/mL)** | **% α-glucosidase**  **inhibition** | **IC50 (µg/mL)**  **α-glucosidase** | **% α-amylase**  **inhibition** | **IC 50 (µg/mL)**  **α-amylase** |
| --- | --- | --- | --- | --- | --- | --- |
|  |  |  | **Mean ± SEM** |  | **Mean ± SEM** |  |
| 1 | Essential oil | 1000  500  250  125  62.5  31.05 | 75.25±0.77***  69.61±0.61***  60.56±0.52***  52.51±1.01***  32.74±0.68***  30.61±0.63*** | 120 | 88.30±0.81***  79.85±0.55***  74.82±0.75***  52.51±1.00***  41.39±0.69***  36.24±0.61*** | 110 |
| 2 | Acarbose | 1000  500  250  125  62.5  31.05 | 91.33±0.33  88.65±0.54  79.01±0.45  72.37±0.61  64.62±0.39  52.36±0.57 | 28 | 90.63±0.99  83.82±0.60  78.31±0.34  70.59±0.26  63.44±0.86  50.86±0.44 | 30 |

The data is represented as mean ± SEM, (n = 3). Values are significantly different as compare to positive control Acarbose, (***P < 0.001).
